# Supplementary figures and images for: Using Known Births to Account for Delayed Marking in Population Estimation of North Atlantic Right Whales
Source: Ecol Evol. 2025 Mar 4;15(3):e71035. doi: 10.1002/ece3.71035 (PMC11879271; doi:10.1002/ece3.71035)

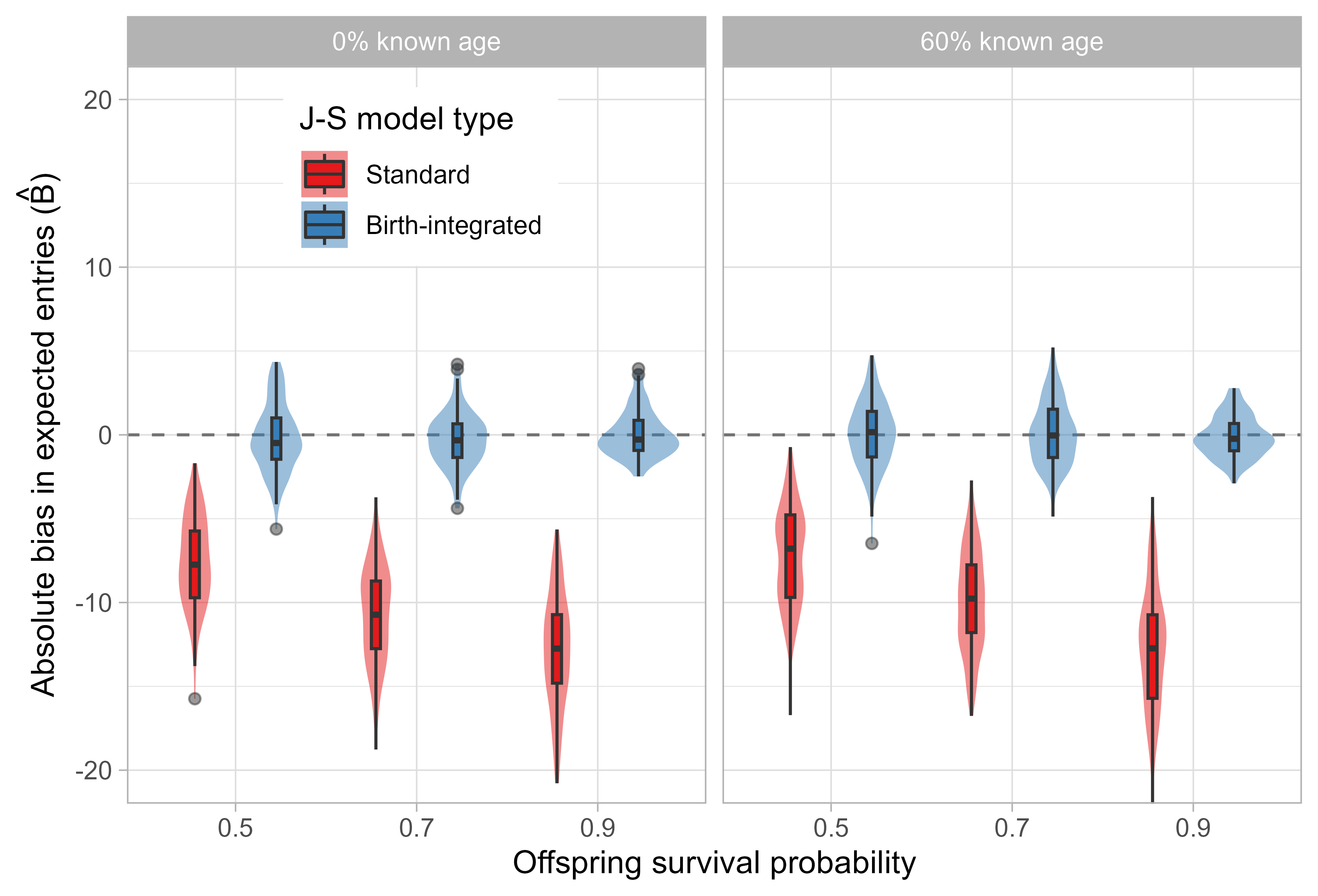

Supplement: Supplementary file 1 — Figure S1. [file ECE3-15-e71035-s002.png]
