# Supplementary material for: Using Known Births to Account for Delayed Marking in Population Estimation of North Atlantic Right Whales
Source: Ecol Evol. 2025 Mar 4;15(3):e71035. doi: 10.1002/ece3.71035 (PMC11879271; doi:10.1002/ece3.71035)
Supplement: Supplementary file 2 — Appendix S1. [file ECE3-15-e71035-s001.pdf]

Appendix

Table S1: Mean relative bias (RB) and root mean square error (RMSE) for estimates of survival (ϕ) and capture probability (p) from the standard and birth-integrated Jolly-Seber (J-S) models fit to simulated capture-recapture data. Simulation scenarios varied by percentage of known-age individuals and the specified probability of offspring mortality (κ).

			$\hat{\phi}$		$\hat{p}$	
J-S Model	Known age (%)	κ	RB	RMSE	RB	RMSE
standard	0	0.1	0.000	0.00	0.000	0.01
standard	0	0.3	0.000	0.00	0.000	0.01
standard	0	0.5	0.000	0.00	0.001	0.01
standard	60	0.1	0.001	0.00	0.001	0.01
standard	60	0.3	0.001	0.00	0.001	0.01
standard	60	0.5	0.001	0.00	0.002	0.01
birth-integrated	0	0.1	0.000	0.00	0.001	0.01
birth-integrated	0	0.3	0.000	0.00	0.000	0.01
birth-integrated	0	0.5	0.000	0.00	0.001	0.01
birth-integrated	60	0.1	0.001	0.00	0.001	0.01
birth-integrated	60	0.3	0.001	0.00	0.001	0.01
birth-integrated	60	0.5	0.001	0.00	0.002	0.01

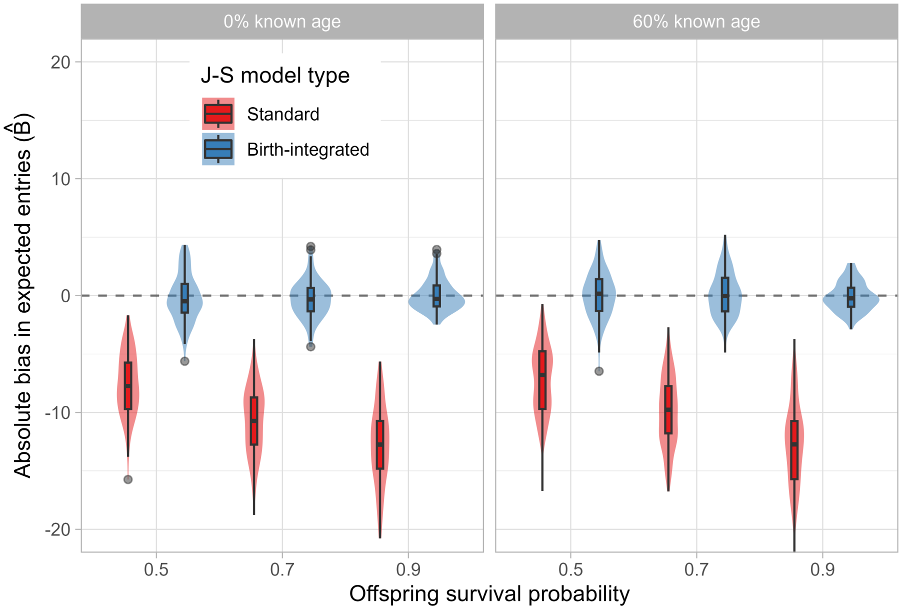

Figure S1: Distributions of absolute bias for estimates of terminal-year entries ($\hat{B_T}$) from the standard and birth-integrated Jolly-Seber (J-S) models fit to simulated capture-recapture data. Simulation scenarios varied by percentage of known-age individuals and the specified probability of offspring mortality (κ), here represented as survival ($1 - \kappa$).

Table S2: Mean and standard deviation of the coefficients of determination (R^2) between actual entries (B_t) and estimated entries ($\hat{B}_t$) from the standard and birth-integrated Jolly-Seber (J-S) models fit to simulated capture-recapture data. Simulation scenarios varied by percentage of known-age individuals and the specified probability of offspring mortality (κ). Comparions excluded the first year ($t = 1$) and terminal year ($t = 20$).

			$\hat{N}_t$		$\hat{B}_t$	
J-S Model	Known age (%)	κ	R^2 mean	R^2 SD	R^2 mean	R^2 SD
standard	0	0.1	0.82	0.14	0.52	0.19
standard	0	0.3	0.90	0.09	0.49	0.21
standard	0	0.5	0.98	0.01	0.49	0.22
standard	60	0.1	0.92	0.07	0.81	0.10
standard	60	0.3	0.93	0.07	0.81	0.11
standard	60	0.5	0.99	0.01	0.79	0.11
birth-integrated	0	0.1	0.87	0.11	0.79	0.11
birth-integrated	0	0.3	0.93	0.07	0.75	0.12
birth-integrated	0	0.5	0.99	0.01	0.68	0.15
birth-integrated	60	0.1	0.93	0.06	0.86	0.07
birth-integrated	60	0.3	0.94	0.07	0.86	0.09
birth-integrated	60	0.5	0.99	0.01	0.83	0.09

Table S3: Posterior distributions of estimated North Atlantic right whale population size using both standard and birth-integrated Jolly-Seber (J-S) models. Old model estimates using sightings data that were available at the time of estimation, while the current model estimates are from 2023.

		Standard J-S (old)		Birth-integrated J-S (old)		Standard J-S (current)	
Year	Calves	N_t median	95% CI	N_t median	95% CI	N_t median	95% CI
2019	7	370	(359,381)	375	(362,387)	378	(376,382)
2020	10	337	(325,350)	345	(331,360)	356	(352,360)
2021	18	341	(333,350)	358	(347,370)	364	(360,369)

Code

R code for the birth-integrated Jolly-Seber model, compatible with BUGS/JAGS/NIMBLE.

```
model {
  p ~ dbeta(1,1)      #capture probability
  phi ~ dbeta(1,1)    #survival probability
  kappa ~ dbeta(1,1)  #offspring loss probability
  # Prior for entry probabilities
  gamma[1] ~ dbeta(1,1)
  for (t in 2:(n.occasions - 1)) {
    # Entry a function of observed births and offspring loss
    gamma[t] <- (births[t]*(1-kappa)) / (M*prod(1-gamma[1:(t-1)]))
  } #t
  # Define state-transition and observation matrices
  # Given state S(t), define probabilities of S(t+1)
  for (t in 1:(n.occasions - 1)) {
    ps[1,t,1] <- 1 - gamma[t]
    ps[1,t,2] <- gamma[t]
    ps[1,t,3] <- 0
    ps[2,t,1] <- 0
    ps[2,t,2] <- phi
    ps[2,t,3] <- 1 - phi
    ps[3,t,1] <- 0
    ps[3,t,2] <- 0
    ps[3,t,3] <- 1
  } #t
  po[1] <- 0
  po[2] <- p
  po[3] <- 0
  # Likelihood
  for (i in 1:M) {
    # Define latent state at first occasion.
    z[i, 1] ~ dcat(px[1:3])   # all M individuals are known z=1 at t=1
    for (t in 2:n.occasions) {
      # State process: draw S(t) given S(t-1)
      z[i,t] ~ dcat(ps[z[i,t-1],t-1,1:3])
      # Observation process: draw O(t) given S(t)
      # NOTE: entries in year t=T have p=0
      y[i,t] ~ dbern(po[z[i,t]]*(1-equals(z[i,n.occasions - 1], 1)))
    } #t
  } #i
  # Derived parameters
  for (i in 1:M) {
    for (t in 1:(n.occasions - 1)) {
      al[i,t] <- equals(z[i,t+1], 2)
      ent[i,t] <- equals(z[i,t] - al[i,t], 0)
    } #t
  } #i
```

```
  for (t in 1:(n.occasions - 1)) {
    N[t] <- sum(al[1:M,t])
    B[t] <- sum(ent[1:M,t])          # Number of entries
  } #t
}
```
